# Heme Oxygenase-1 Is Involved in the Repair of Oxidative Damage Induced by Oxidized Fish Oil in *Litopenaeus vannamei* by Sulforaphane

**DOI:** 10.3390/md21100548

**Published:** 2023-10-23

**Authors:** Junliang Luo, Yongxiong Huang, Yanghui Chen, Yunhao Yuan, Guojian Li, Shuanghu Cai, Jichang Jian, Shiping Yang

**Affiliations:** Guangdong Provincial Key Laboratory of Aquatic Animal Disease Control and Healthy Culture & Key Laboratory of Control for Disease of Aquatic Animals, Guangdong Higher Education Institutes, Fisheries College, Guangdong Ocean University, Zhanjiang 524088, China; 2112101032@stu.gdou.edu.cn (J.L.); yingxiongh788@gmail.com (Y.H.); chenyanghui11@stu.gdou.edu.cn (Y.C.); 15837688713@163.com (Y.Y.); liguojian282436@163.com (G.L.); caish@gdou.edu.cn (S.C.); jianjc@gdou.edu.cn (J.J.)

**Keywords:** *Litopenaeus vannamei*, sulforaphane, *HO-1*, oxidative damage, apoptosis, autophagy

## Abstract

Heme oxygenase-1 (HO-1), which could be highly induced under the stimulation of oxidative stress, functions in reducing the damage caused by oxidative stress, and sulforaphane (SFN) is an antioxidant. This study aims to investigate whether *HO-1* is involved in the repair of oxidative damage induced by oxidized fish oil (OFO) in *Litopenaeus vannamei* by sulforaphane (SFN). The oxidative stress model of *L. vannamei* was established by feeding OFO feed (OFO accounts for 6%), and they were divided into the following four groups: control group (injected with dsRNA-EGFP and fed with common feed), dsRNA-*HO-1* group (dsRNA-*HO-1*, common feed), dsRNA-*HO-1* + SFN group (dsRNA-*HO-1*, supplement 50 mg kg^−1^ SFN feed), and SFN group (dsRNA-EGFP, supplement 50 mg kg^−1^ SFN feed). The results showed that the expression level of *HO-1* in the dsRNA-*HO-1* + SFN group was significantly increased compared with the dsRNA-*HO-1* group (*p* < 0.05). The activities of SOD in muscle and GPX in hepatopancreas and serum of the dsRNA-*HO-1* group were significantly lower than those of the control group, and MDA content in the dsRNA-*HO-1* group was the highest among the four groups. However, SFN treatment increased the activities of GPX and SOD in hepatopancreas, muscle, and serum and significantly reduced the content of MDA (*p* < 0.05). SFN activated *HO-1*, upregulated the expression of antioxidant-related genes (*CAT*, *SOD*, *GST*, *GPX*, *Trx*, *HIF-1α*, *Nrf2*, *prx 2*, *Hsp 70*), and autophagy genes (*ATG 3*, *ATG 5*), and stabilized the expression of apoptosis genes (*caspase 2*, *caspase 3*) in the hepatopancreas (*p* < 0.05). In addition, knocking down *HO-1* aggravated the vacuolation of hepatopancreas and increased the apoptosis of hepatopancreas, while the supplement of SFN could repair the vacuolation of hepatopancreas and reduce the apoptosis signal. In summary, *HO-1* is involved in the repair of the oxidative damage induced by OFO in *L. vannamei* by SFN.

## 1. Introduction

*Litopenaeus vannamei* is one of the most widely cultivated prawn species in China, with fast growth and high economic value. However, shrimp often suffer from various oxidative stresses in the aquaculture environment, such as ammonia and nitrite nitrogen stimulation [1], abnormal salinity stimulation [2], etc., which have a negative impact on the body’s antioxidant and immune abilities, eventually leading to the outbreak of shrimp diseases and restricting the healthy development of shrimp aquaculture. Oxidative stress reflects the imbalance between reactive oxygen species (ROS) produced and accumulated in cells and tissues and the body’s anti-stress ability [3]. Excessive accumulation of free radicals in the body will not only damage macromolecules such as DNA and protein but also lead to lipid peroxidation [4]. Fish oil contains unsaturated fatty acids, which are essential for the growth and reproduction of aquatic animals [5]. Fish oil, as one of the important ingredients in shrimp feed, is easily oxidized to oxidized fish oil (OFO), which can cause oxidative stress in shrimp. OFO is often used to establish the oxidative stress model of aquatic animals because it has the negative effect of reducing the antioxidant capacity of aquatic animals, leading to liver injury and fatty liver in aquatic animals [6,7].

Heme oxygenase-1 (HO-1), which could be highly induced by the stimulation of oxidative stress, has the function of reducing the damage caused by oxidative stress [8]. Previous studies have shown that *HO-1* could alleviate cell stress and injury by reducing inflammation, regulating antioxidation, and inhibiting apoptosis [9,10]. Stimulated by lipopolysaccharide, mice that knock down *HO-1* will suffer more oxidative stress, resulting in increased mortality and end organ damage [11]. Hypoxia increases the activity of *HO-1* in fish gills and increases the acute hypoxic ventilation frequency response after inhibition of *HO-1* [12].

Sulforaphane (SFN), as a natural antioxidant, has strong antioxidant performance and a significant role in stabilizing free radicals [13]. SFN could alleviate the stress on the endoplasmic reticulum of hippocampal neurons caused by a high glucose environment, reduce neuronal apoptosis [14], significantly reduce the oxidative damage of *L. vannamei* stimulated by ammonia nitrogen, and improve its antioxidant capacity [15]. SFN can not only reduce the content of malondialdehyde (MDA) and improve antioxidant capacity, but also reduce the production of ROS, thus reducing myocardial cell damage caused by ischemia/reperfusion [16,17].

Based on the above research, the purpose of this study is to evaluate the repair effect of SFN and explore whether *HO-1* is involved in the repair of oxidative damage induced by OFO in *L. vannamei* by SFN, to further understand the protective effect of SFN and the function of *HO-1* in oxidative stress.

## 2. Result

### 2.1. Expression Profile of HO-1 after Knock-Down

**Figure 1:**
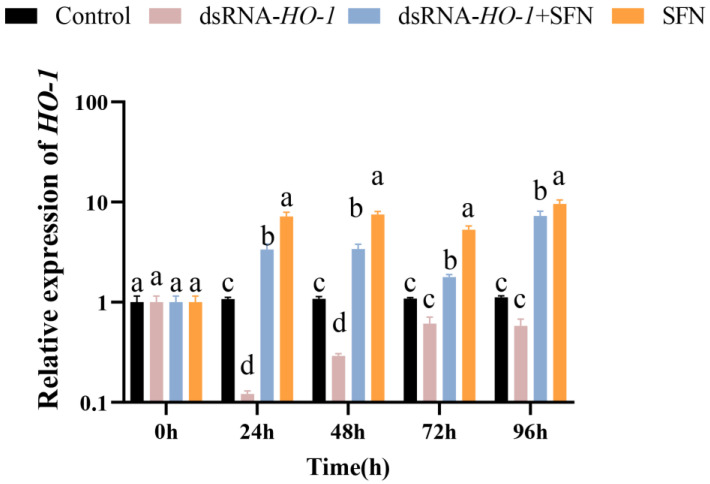
The expression of *HO-1* in the hepatopancreas of four groups. Different letters denote a significant difference (*p* < 0.05).

As shown in Figure 1, in the hepatopancreas, the expression of *HO-1* in the dsRNA-*HO-1* + SFN and SFN groups increased significantly compared with the control and dsRNA-*HO-1* groups (*p* < 0.05). The expression of *HO-1* in the SFN group was significantly higher than that in the control group (*p* < 0.05).

### 2.2. Determination of Antioxidative Parameters

**Figure 2:**
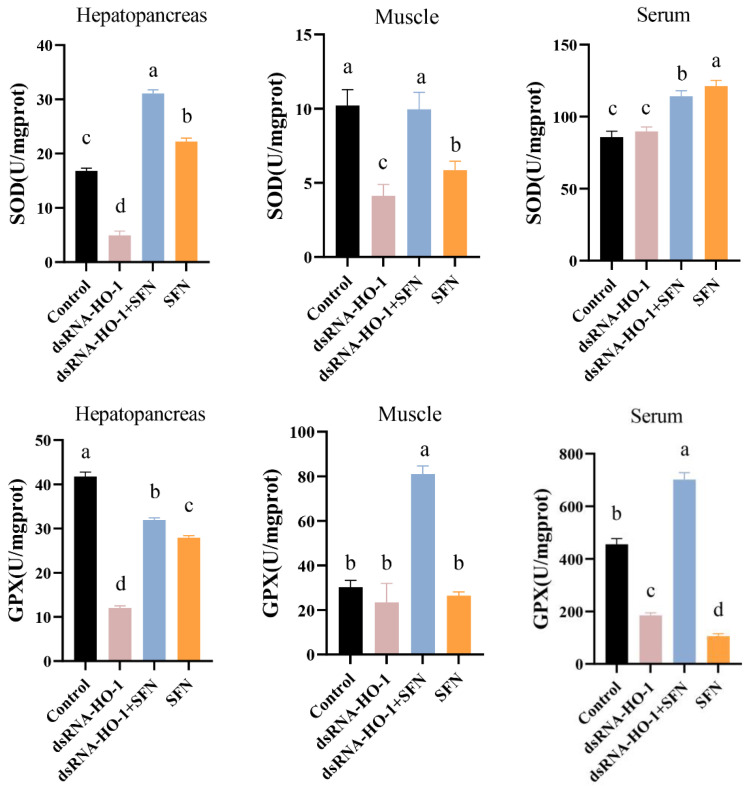

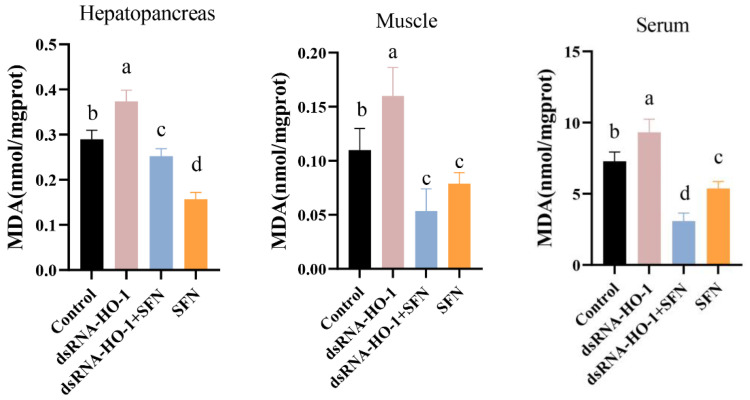
The activities of SOD, GPX, and the MDA content in the hepatopancreas, muscle, and serum of *L. vannamei*. Different letters denote a significant difference (*p* < 0.05).

As shown in Figure 2, the activities of GPX in the muscle and serum of the dsRNA-*HO-1* + SFN group markedly increased compared with those of the control and dsRNA-*HO-1* groups (*p* < 0.05). In the dsRNA-*HO-1* group, the activities of SOD in the muscle and hepatopancreas and GPX in the serum significantly decreased compared with those of the control and dsRNA-*HO-1* + SFN groups (*p* < 0.05). The content of MDA in the hepatopancreas, muscle, and serum was the highest in the dsRNA-*HO-1* group, while it was lower in the dsRNA-*HO-1* + SFN and SFN groups (*p* < 0.05).

### 2.3. Expression of Antioxidant-Related Genes

**Figure 3:**
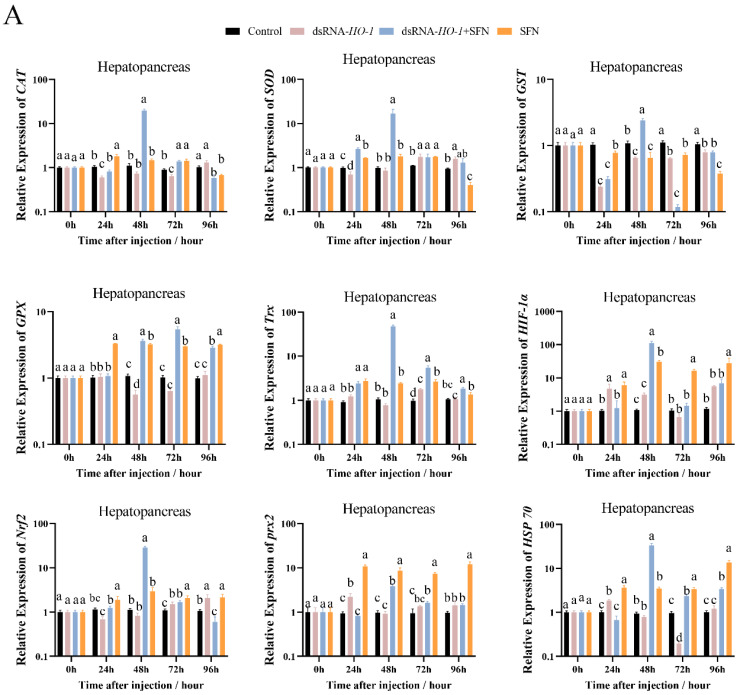

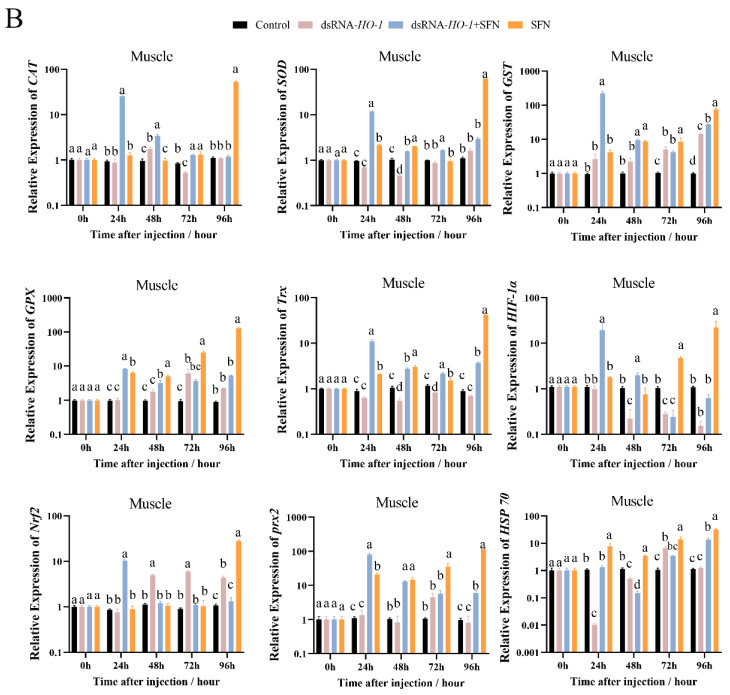
The expression of antioxidant-related genes (*CAT*, *SOD*, *GST*, *GPX*, *Trx*, *HIF-1α*, *Nrf2*, *prx 2*, and *Hsp 70*) in hepatopancreas (**A**) and muscle (**B**). Different letters denote a significant difference (*p* < 0.05).

As shown in Figure 3A, at 48 h, the expression levels of *GPX*, *prx 2*, and *HSP 70* were significantly increased in the dsRNA-*HO-1* + SFN and SFN groups compared with the dsRNA-*HO-1* group (*p* < 0.05). In the hepatopancreas, the expression of antioxidant-related genes in the dsRNA-*HO-1* + SFN group basically reached its peak at 48 h, and the levels of *CAT*, *SOD,* and *GST* in the dsRNA-*HO-1* group were significantly lower than those in the control group at 24 h (*p* < 0.05). In muscle, the expression levels of antioxidant-related genes in the SFN group showed an upward trend from 0 to 96 h, and the peak expression of these genes was concentrated at 96 h.

### 2.4. Expression of Apoptosis- and Autophagy-Related Genes

**Figure 4:**
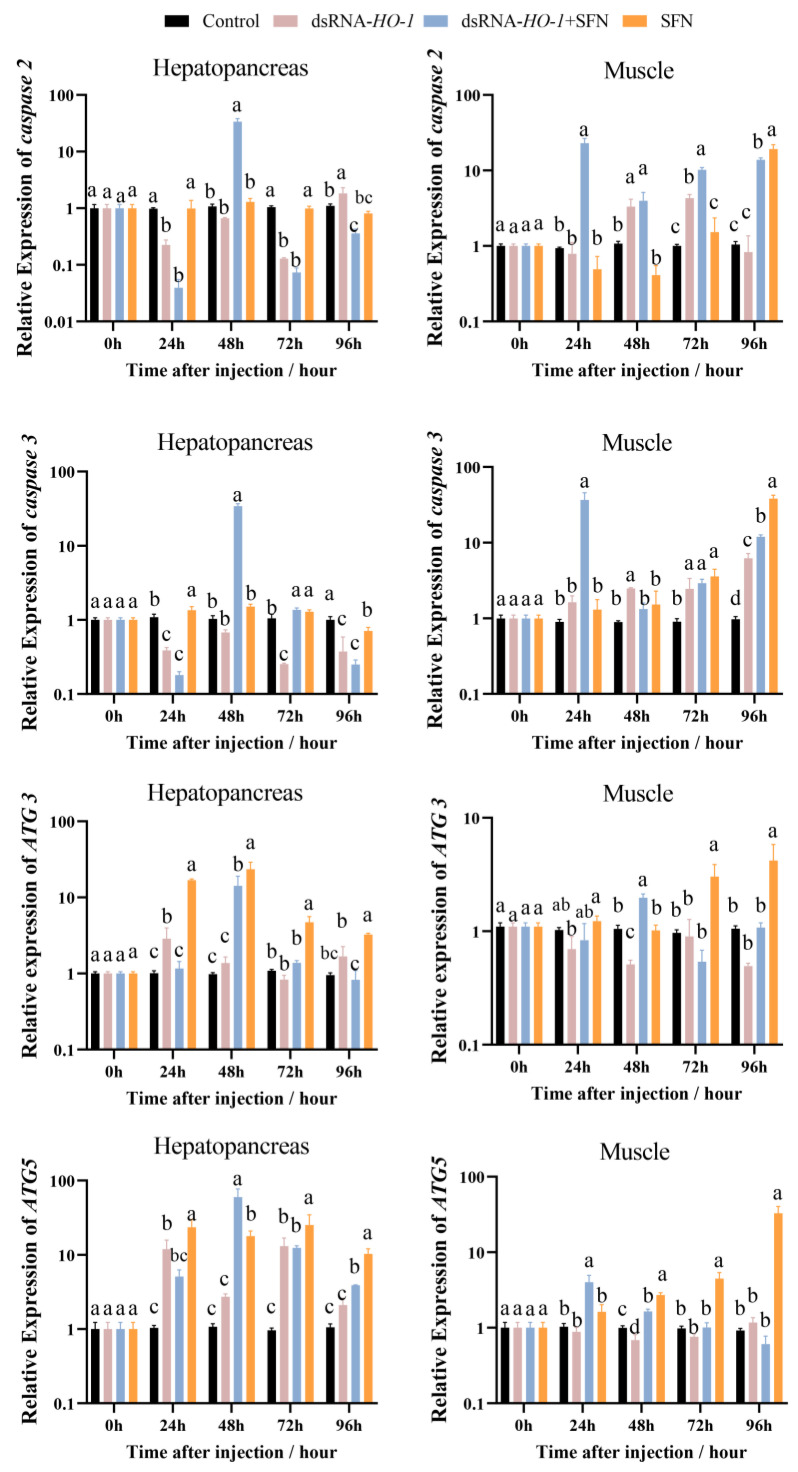
The expression of apoptosis-related and autophagy-related genes (*caspase 2*, *caspase 3*, *ATG 3*, and *ATG 5*) in the hepatopancreas and muscle. Different letters denote a significant difference (*p* < 0.05).

In the hepatopancreas, the expression of *caspase 2* and *caspase 3* in the dsRNA-*HO-1* + SFN group first decreased, then increased, and then decreased from 0 to 96 h. At 96 h, the expression of *caspase 2* in the dsRNA-*HO-1* group was the highest among the four groups (*p* < 0.05, Figure 4). The expression peaks of *ATG 3* and *ATG 5* in the dsRNA-*HO-1* + SFN group appeared at 48 h (*p* < 0.05). In muscle, the expression of *caspase 2* in the dsRNA-*HO-1* group first increased and then decreased from 24 to 96 h. Moreover, the expression of *ATG 5* in the dsRNA-*HO-1* + SFN group decreased gradually after reaching the peak at 24 h, while that in the SFN group increased gradually from 24 h to 96 h (*p* < 0.05).

### 2.5. Hepatopancreatic Histology

**Figure 5:**
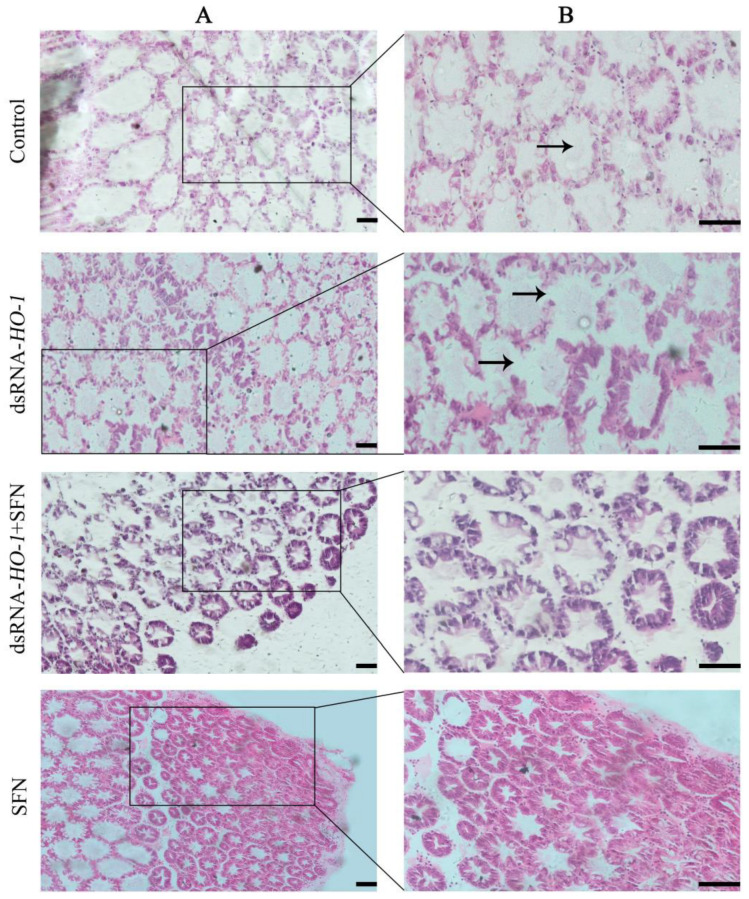
Histological analysis of hepatopancreas in four groups. Hematoxylin and eosin staining under 100× (**A**) and 200× (**B**) magnifications. The position shown by the arrow represents the cavitation and wall thinning of the tubule (Scale plate: 50 μm).

As shown in Figure 5, the pathological sections of the hepatopancreas of the oxidized model shrimp in the control group showed an abnormal structure, including vacuolation of the lumen and obvious enlargement of some lumens. In the dsRNA-*HO-1* group, not only the lumen was vacuolated, but also the wall of the tube was thinned. However, in the dsRNA-*HO-1* + SFN and SFN groups, the lumen cavity and abnormal wall were improved, and the lumen was star-shaped.

### 2.6. Detection of Hepatopancreatic Apoptosis

**Figure 6:**
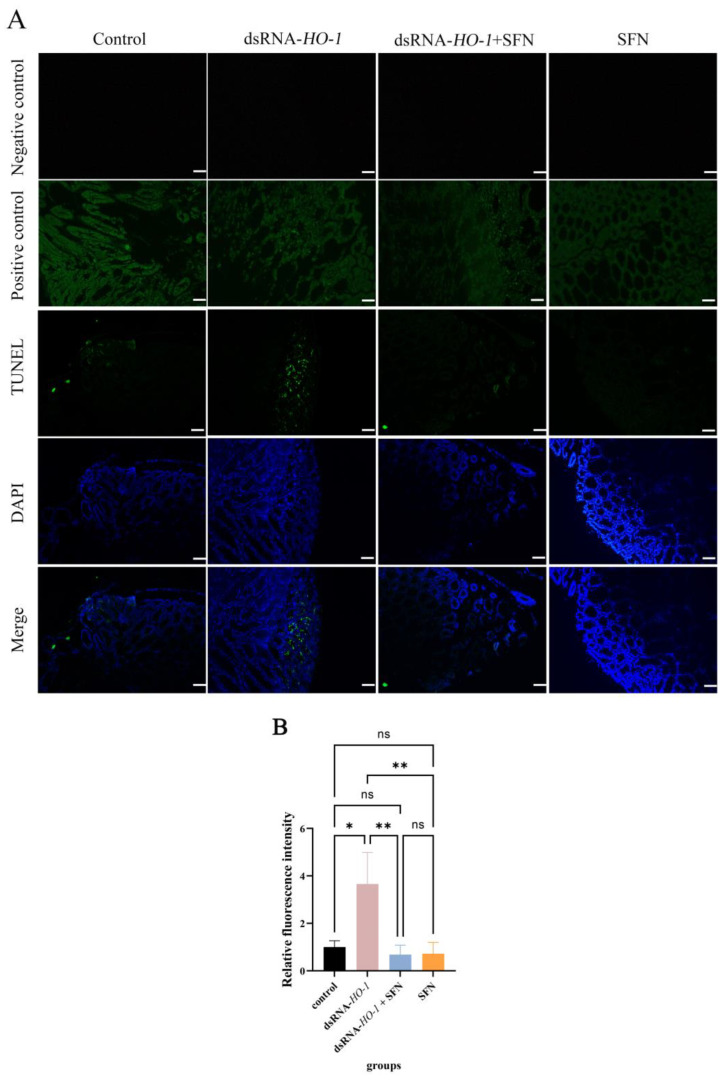
Apoptosis of hepatopancreatic cells of *L. vannamei*. The signal of apoptosis is marked in green. The nucleus is marked in blue. All sections were observed at 100× magnification (**A**), and the relative fluorescence intensity of four groups was compared (**B**) (Scale plate: 50 μm). The asterisks reveal a significant difference (“*” means that *p* < 0.05, “**” means that *p* < 0.01, ns: no significance).

As shown in Figure 6, in the control group and the dsRNA-*HO-1* group, an obvious green fluorescent signal was observed, and the signal was stronger in the dsRNA-*HO-1* group. However, this signal was significantly attenuated in the dsRNA-*HO-1* + SFN group. In the positive control group, the apoptotic TUNEL-positive nuclei were digested by DNase 1 and labeled with FITC to show green fluorescence.

## 3. Materials and Methods

### 3.1. OFO and Experimental Diets

Fresh fish oil with a peroxide value (POV) of 1.15 meq kg^−1^ was placed in a water bath pot at 55 °C and continuously oxygenated with an air pump until the POV was 120 meq kg^−1^. Then, OFO was placed at −20 °C to prevent further oxidation until use. In addition, shrimp feed was purchased from Guangdong Yuehai Feed Group Co., Ltd. (Zhanjiang, China), and SFN was purchased from Aladdin Company (Shanghai, China). Commercial feed contains approximately 43% crude protein, 5% crude fat, and 16% crude ash. Two experimental diets were prepared for shrimp: (1) a supplemental diet with OFO and without SFN (OFO feed), used for constructing oxidative stress models; and (2) a supplemental diet with OFO and SFN (with 50 mg kg^−1^ SFN addition, SFN feed), used for later experiments. The experimental diet was mixed evenly and stored at −20 °C to prevent its components from deteriorating.

### 3.2. Construction of an Oxidative Stress Model

*L. vannamei* juveniles were provided by a breeding hatchery (Zhanjiang, China). Before the experiment, they were raised in an outdoor cement pool for 20 days, and then shrimp with clear body color, good vitality, and a clear and full outline of the stomach, hepatopancreas, and intestine were randomly divided into four groups (the group name will be named in 2.3) with three parallel barrels at a density of 30 shrimp per barrel. After 10 days of acclimation, shrimp (the average weight was 0.75 ± 0.05 g) were fed with the OFO feed (OFO accounts for 6% of the feed; the daily feeding amount is 10% of the total weight of shrimp) prepared above at 8:00, 12:00, 16:00, and 22:00 for 28 consecutive days [18]. The feces and leftover diets were removed by syphoning. At the same time, two-thirds of the water in each bucket was changed every two days, and air was continuously injected into the water in the bucket during this period. During the experimental period, the water temperature was maintained at 26.8–28.0 °C, and the pH and salinity values were maintained at 8.0–8.2 and 26.7‰, respectively.

### 3.3. RNAi Assay

According to previous research [19], the double-stranded RNA (dsRNA) of enhanced green fluorescent protein (EGFP) and *HO-1* was synthesized with the T7 RNAi transcription kit (Takara, Beijing, China). The quality and quantity of dsRNA were detected by 1% agar gel and spectrophotometry. After being fed with OFO feed for 28 days, the shrimp were starved for 24 h and then injected with dsRNA. For the dsRNA-*HO-1* group, the shrimp were injected with dsRNA-*HO-1* (0.50 μg/g shrimp; the same as below) and fed with common feed (commercial feed). For the dsRNA-*HO-1* + SFN group, the shrimp were injected with dsRNA-*HO-1* and fed with SFN supplement feed. For the control group, the shrimp were injected with dsRNA-EGFP and fed with common feed. For the SFN group, the shrimp were injected with dsRNA-EGFP and fed with SFN supplement feed. The hepatopancreas and muscles of shrimp were sampled at 24, 48, 72, and 96 h after injection (six shrimp were sampled from each group, and the hepatopancreas and muscles of nine shrimp in the control group were taken as control before injection) for total RNA extraction.

### 3.4. Sample Collection

During the experiment, the hemolymph, hepatopancreas, and muscle of five shrimp in each group were randomly collected for enzyme activity determination, and the hepatopancreas and muscle of six shrimp in each group were randomly collected for the qRT-PCR experiment. Meanwhile, the hepatopancreas of six shrimp in each group were also randomly collected and fixed with Carnoy’s Fluid for pathological analysis and TUNEL apoptosis detection. After hemolymph was collected, it was left at 4 °C for 12 h, then centrifuged at 3500 rpm at 4 °C for 10 min, and the supernatant was obtained. The supernatant was stored at −80 °C until it was used for enzyme activity determination. All shrimp were anesthetized before sampling.

### 3.5. Histological Analysis

Samples of hepatopancreas were fixed in Carnoy’s Fluid for 24 h, dehydrated in gradient ethanol (70%, 85%, 95%, and 100%), made transparent in xylene, embedded in paraffin, and sliced into 8 μm sections. After dewaxing and rehydrating, the slices were stained with a hematoxylin and eosin staining kit (Beyotime, Shanghai, China). The stained sections were observed and photographed with a Nikon DS-Ri2 microscope (Nikon, Tokyo, Japan).

### 3.6. TUNEL Apoptosis Detection

The apoptosis in the hepatopancreas was measured by a TUNEL assay kit (Green FITC, Elabscience, Wuhan, China). According to the manufacturer’s instructions, the paraffin sections prepared above were used for testing.

### 3.7. Measurement of Biochemical Parameters

In accordance with the scheme of the manufacturer, the activities of SOD and GPX and the content of MDA were analyzed using a commercial assay kit (Nanjing Jiancheng Bioengineering Institute, Nanjing, China).

### 3.8. Determination of mRNA Expression

Total RNA was immediately extracted from hepatopancreas and muscle by using RNAiso Plus (TaKaRa, Dalian, China). The quality and quantity of the extracted RNA were evaluated in the same manner as described above. The PrimeScript RT kit (Takara, Dalian, China) containing a gDNA eraser was used to reverse-transcribe the extracted total RNA.

The expressions of genes related to antioxidation, autophagy, and apoptosis in the hepatopancreas and muscle of *L. vannamei* were detected using the Quant Studio 6 Flex RT-PCR system (Thermo Fisher Scientific, Waltham, MA, USA) and PerfectStart Green qPCR SuperMix (Trans). The *EF-1 α* gene of *L. vannamei* was used as the internal control. Table 1 lists all the primer sequences used in this study. The relative gene expression level of the data was analyzed by the 2^−ΔΔCt^ method [20]. All data were presented as mean ± S.E.M from three samples with three parallel repetitions.

### 3.9. Statistical Analysis

The data of each group were analyzed by one-way ANOVA with SPSS software (SPSS 18.0; SPSS, Chicago, IL, USA), and different lowercase letters showed significant differences between the groups, and the significance level was set to *p* < 0.05.

## 4. Discussion

Our previous research successfully cloned the *HO-1* gene of *L. vannamei*, detected its expression in different tissues, and found that it participated in the antioxidant and anti-apoptosis effects of ammonia-induced oxidative stress [19]. In this study, we found that SFN could repair the oxidative damage caused by OFO to *L. vannamei* and significantly improve the expression level of *HO-1* in the hepatopancreas and muscles. These preliminary results indicated that SFN could repair oxidative damage by activating *HO-1*.

The members of the antioxidant system, including CAT, SOD, GST, and GPX, are usually used as indicators to evaluate the current antioxidant status of the organism [21]. Antioxidant enzymes are usually upregulated after cells are exposed to oxidative stress to reduce damage caused by oxidative stress [22,23]. In this study, SFN supplementation significantly increased the activities of GPX and SOD in shrimp (*p* < 0.05). These results indicated that SFN may be beneficial for improving the antioxidant capacity of *L. vannamei*. This is consistent with the result that the antioxidant enzyme activity in shrimp tissues increased significantly compared with the control group after eating SFN-supplemented feed [15]. *Trx* plays an important role in maintaining the balance between oxidative stress and the antioxidant system and protecting the body from oxidative damage [24]. *HIF-1α* can rapidly induce the expression of genes related to oxygen utilization, thus improving the oxygen utilization of cells [25], and *prx2* is a member of peroxidase [26]. Under the stimulation of copper-induced oxidation, the Nrf2/HO-1 pathway was activated, thus alleviating the damage caused by oxidative stress [8]. Overexpression of *HO-1* will not only weaken the replication of the hepatitis C virus but also protect liver cells from oxidative damage [27]. In this study, compared with those of the control group and dsRNA-*HO-1* group, the expression levels of *CAT*, *SOD*, *GST*, *GPX*, *Trx*, *HIF-1α*, *prx 2,* and *HSP 70* in the hepatopancreas and muscle of the dsRNA-*HO-1* + SFN group were significantly increased (*p* < 0.05). This may be because SFN treatment activates the Nrf2/HO-1 pathway, thus regulating the expression of a series of antioxidant-related genes and finally alleviating oxidative damage caused by oxidative stress. *HSP 70* could be activated by oxidative stress, similar to *HO-1*, and could reduce the damage caused by oxidative stress [28,29]. In this study, SFN activated the expression of antioxidant-related genes, including *HO-1* and *HSP 70*, and enabled the body to better repair the damage caused by OFO.

Oxidative stress can cause cell apoptosis [30]. HO in the nervous system of *Drosophila melanogaster* is closely related to apoptosis [31]. In this study, compared with the control group, the expression levels of *caspae 2* and *caspase 3* in the hepatopancreas of the dsRNA-*HO-1* + SFN group reached their peak at 48 h and were then lower than those in the control group at 96 h (*p* < 0.05). Furthermore, there was no significant difference in the expression level of *caspase 2* between the SFN group and the control group. However, at 96 h, the expression of *caspase 2* in the dsRNA-*HO-1* group was the highest among the four groups (*p* < 0.05). The TUNEL experiment also showed that when *HO-1* was knocked down, there would be more apoptosis signals in the hepatopancreas, but this apoptosis signal was obviously weakened after SFN treatment. Overexpression of *HO-1* inhibited the apoptosis of bovine ovarian granulosa cells [32]. In addition, Cobalt-protoporphyrin could reduce liver injury by increasing the expression of *HO-1,* and Sichuan pepper could also enhance its antioxidant defense system by upregulating the expression of *HO-1* [33,34]. These results confirmed that the treatment of SFN increased the expression of *HO-1*, and *HO-1* participated in the process of regulating apoptosis and was finally involved in the repair of oxidative damage by SFN. Interestingly, the expression level of *caspase 3* in the hepatopancreas of the dsRNA-*HO-1* group was also lower than that of the control group at 96 h, and it was basically the same as that of the dsRNA-*HO-1* + SFN group. The reason may be that cells are necrotic due to excessive oxidative damage, thus reducing the expression of apoptosis and apoptosis-related genes.

Autophagy is an evolutionary-conserved intracellular process that is used to degrade and recycle cellular materials [35]. *HO-1* can induce protective autophagy and reduce emphysema caused by cadmium [36]. Overexpression of *HO-1* can significantly restore autophagy and protect cells from apoptosis caused by the external environment [37]. This study also found that SFN increased autophagy in the hepatopancreas and muscle by increasing the expression level of *HO-1* and finally reducing cell apoptosis. These results indicated that *HO-1* could repair the oxidative damage induced by OFO by promoting autophagy.

Hepatopancreas is the most important organ in crustaceans and is extremely sensitive to pollutants in the diet, so hepatopancreas is usually used to monitor the effects of various poisons on the body [38]. Knocking down *HO-1* in *L. vannamei* will significantly change the morphology of hepatic tubules [19]. This study also found similar results. After knocking down *HO-1*, the wall of hepatic tubules became thinner or even disappeared. However, this phenomenon was restored in the dsRNA-*HO-1* + SFN group. The existence of astaxanthin significantly improved the abnormal tubular structure and arrangement of the hepatopancreas caused by the consumption of OFO by *L. vannamei* [39]. MDA is usually used as a symbol of oxidative stress. In this study, the MDA content in the dsRNA-*HO-1* group was the highest, while that in the dsRNA-*HO-1* + SFN group was the lowest (*p* < 0.05). These results showed that when the expression of *HO-1* was inhibited, the body would suffer from more oxidative stress, which could lead to more serious oxidative damage. SFN could promote the expression of *HO-1*, which could help the body recover from oxidative damage more quickly. The increase in *HO-1* expression levels reduces oxidative damage and the production of ROS [40]. The results showed that SFN could repair oxidative damage caused by OFO by promoting the expression of *HO-1*.

In summary, SFN could increase the antioxidant enzyme activity and reduce the MDA content in the hepatopancreas, muscle, and serum of *L. vannamei*. SFN could activate the expression of *HO-1* in *L. vannamei*, thus regulating the expression of antioxidant, autophagy, and apoptosis genes and finally repairing the oxidative damage brought by OFO on *L. vannamei*. Knocking down *HO-1* aggravated the vacuolation of hepatopancreas and increased the apoptosis of hepatopancreas, and the existence of SFN could repair the oxidative damage of hepatopancreas and reduce the apoptosis signal. The results indicated that *HO-1* is involved in the repair of oxidative damage induced by OFO in *L. vannamei* by SFN.

## Figures and Tables

**Table 1:** Sequences of primers used in this study.

Primer Name	Sequence (5′–3′)	GenBank Accession Number	Product Length
qHO-1-F	GCATGGCAGTGACCGAGATTGA	XM_027376282.1	108
qHO-1-R	GTCGCTGCTTCGTCTCCTCATC		
qCAT-F	TCAGCGTTTGGTGGAGAA	AY518322.1	147
qCAT-R	GCCTGGCTCATCTTTATC		
qNrf2-F	GATGAGAAGCGAGCCAGAGCG	XM_027367068.1	142
qNrf2-R	GCCGTCGGATGTCTCGGATAA		
qHSP70-F	GCGTACTGCCTGTGAGCG	AY645906	108
qHSP70-R	CGGGTGATGGAGGTGTAGAAA		
qGST-F	AAGATAACGCAGAGCAAGG	AY573381.2	146
qGST-R	TCGTAGGTGACGGTAAAGA		
qGPX-F	AGGGACTTCCACCAGATG	XM_027372127.1	117
qGPX-R	CAACAACTCCCCTTCGGTA		
qSOD-F	CTGGTTCCGTTGCTTGGC	DQ005531	122
qSOD-R	CGCTCATTCACGTTCTCCC		
qTrx-F	TTAACGAGGCTGGAAACA	XM_027377405.1	116
qTrx-R	AACGACATCGCTCATAGA		
qHIF-1*α*-F	GGAGGCCTACAAGACACTGC	FJ807918.1	152
qHIF-1*α*-R	TGAGACACACGACGTACTGC		
qprx2-F	AATGACCGCGTTGAGGAGTT	XM_027353910.1	134
qprx2-R	AGTGGGATCTTCAGCTTGCC		
qATG3-F	CGCTGCCAAGACCAAACCATA	MH797018.1	105
qATG3-R	TGCTCACTGCGATACTCCATT		
qATG5-F	GGAACCTCACTGCCCACTTT	MH797023.1	127
qATG5-R	TGCCCTCTGTGCTTCAAACC		
qcaspase2-F	TAAAGTTCCCTCACGACAA	XM_027358707.1	278
qcaspase2-R	GCTCATCACCATCCCTAAT		
qcaspase3-F	AACCAAGGCATCCCTGTCA	XM_027378310.1	190
qcaspase3-R	GGGTTTATTCTGAAGTTGTGGG		
qEF1*α*-F	GTATTGGAACAGTGCCCGTG	XM_027373349.1	143
qEF1*α*-R	ACCAGGGACAGCCTCAGTAAG		
